# Multi-modal remote perception learning for object sensory data

**DOI:** 10.3389/fnbot.2024.1427786

**Published:** 2024-09-19

**Authors:** Nouf Abdullah Almujally, Adnan Ahmed Rafique, Naif Al Mudawi, Abdulwahab Alazeb, Mohammed Alonazi, Asaad Algarni, Ahmad Jalal, Hui Liu

**Affiliations:** ^1^Department of Information Systems, College of Computer and Information Sciences, Princess Nourah Bint Abdulrahman University, Riyadh, Saudi Arabia; ^2^Department of Computer Science and IT, University of Poonch Rawalakot, Rawalakot, Pakistan; ^3^Department of Computer Science, College of Computer Science and Information System, Najran University, Najran, Saudi Arabia; ^4^Department of Computer Engineering, College of Computer Engineering and Sciences, Prince Sattam Bin Abdulaziz University, Al-Kharj, Saudi Arabia; ^5^Department of Computer Sciences, Faculty of Computing and Information Technology, Northern Border University, Rafha, Saudi Arabia; ^6^Faculty of Computer Science, Air University, Islamabad, Pakistan; ^7^Department of Computer Science and Engineering, College of Informatics, Korea University, Seoul, Republic of Korea; ^8^Cognitive Systems Lab, University of Bremen, Bremen, Germany

**Keywords:** multi-modal, sensory data, objects recognition, visionary sensor, simulation environment multi-modal, simulation environment

## Abstract

**Introduction:**

When it comes to interpreting visual input, intelligent systems make use of contextual scene learning, which significantly improves both resilience and context awareness. The management of enormous amounts of data is a driving force behind the growing interest in computational frameworks, particularly in the context of autonomous cars.

**Method:**

The purpose of this study is to introduce a novel approach known as Deep Fused Networks (DFN), which improves contextual scene comprehension by merging multi-object detection and semantic analysis.

**Results:**

To enhance accuracy and comprehension in complex situations, DFN makes use of a combination of deep learning and fusion techniques. With a minimum gain of 6.4% in accuracy for the SUN-RGB-D dataset and 3.6% for the NYU-Dv2 dataset.

**Discussion:**

Findings demonstrate considerable enhancements in object detection and semantic analysis when compared to the methodologies that are currently being utilized.

## 1 Introduction

Intelligent systems acquire information about their surroundings by engaging in contextual scene learning, which entails establishing connections among different environmental components. The system collects, evaluates, and interprets visual data from its environment to provide relevant and context-specific clues for understanding the situation. The framework can assess contextual scene learning and identify objects by detecting the spatial and semantic connections between objects and eliminating their features. This will facilitate the transition of systems from understanding to resilience and context awareness. Song et al. (2015) introduced the SUN RGB-D dataset, which provides a comprehensive benchmark for RGB-D scene understanding, highlighting the necessity of multi-modal data for accurate scene interpretation. This work highlights the challenges in integrating multiple data types to enhance object recognition and scene comprehension.

There has been a recent increase in interest in developing computational frameworks that can comprehend the complexities of vast quantities of visual data. Computerized systems with the ability to accurately identify objects and determine their importance in a certain situation are essential for augmented reality, surveillance, and autonomous vehicles (Rabia et al., 2014; Murugan et al., 2022; Ghasemi et al., 2022). Optimal results require innovative approaches to managing several situations within a specific setting.

The current investigation introduces a novel approach known as Deep Fused Networks (DFN) to tackle these issues. DFN, in contrast to traditional approaches, seeks to integrate several sophisticated methodologies by leveraging the capabilities of deep learning to overcome their limitations. The primary objective is to enhance accuracy in identifying many items in challenging conditions. DFN is a reliable framework for object detection that effectively combines several models by emphasizing their distinctive qualities. This novel methodology facilitates the identification of items in intricate scenarios characterized by factors such as partial concealment (occlusion), alterations in size, and crowded backdrops. Moreover, and perhaps most significantly, DFN facilitates a comprehensive comprehension of visual images by analyzing their underlying meaning. This framework utilizes the context of existing objects to extract more advanced information, such as scene characteristics, object relationships, and item categorization within the scene or environment.

Contextual scene understanding has drawn a lot of interest lately because of its vital uses in robotics, autonomous vehicles, and surveillance. Due to the inherent constraints of a single modality, traditional approaches that rely merely on RGB (color), images frequently struggle to appropriately interpret complex scenarios. For example, it can be difficult to discriminate between objects, like a red ball on a red carpet, that have identical colors but different textures or depths. Our proposed approach uses multi-modal data—that is, RGB and depth (RGB-D) information—to overcome these challenges. We can capture both the geometric and visual aspects of the scene by combining RGB images with depth information, which results in a more accurate and robust contextual understanding. By incorporating multi-modality, we have addressed the following important challenges:

- Multi-modal data helps in discriminating objects that appear similar in RGB images but have distinct geometric properties.
- Depth information aids in identifying partially occluded objects, providing a clearer understanding of the scene.
- Combining RGB and depth data enriches the feature set, allowing for better semantic segmentation and scene understanding.

This comprehensive analysis enhances comprehension of the visual context and facilitates more informed decision-making. To evaluate the effectiveness of our proposed DFN framework, we conducted experiments using established benchmark datasets and conducted a comparative analysis with other existing approaches. The results of our studies indicate that the DFN model is both robust and successful since it achieved gains in both multi-object identification accuracy and semantic analysis. To summarize, this research proposes a complete strategy to address the difficulties of achieving dependable multi-object detection and semantic analysis to enhance scene comprehension. The suggested Deep Feature Network (DFN) enhances both the effectiveness of object detection and the comprehension of visual situations, hence creating new opportunities for diverse computer vision applications.

- Deep Fused Network for Scene Contextual Learning through Object Categorization: The study introduces a deep-fused network that facilitates scene contextual learning by incorporating object categorization.
- FuseNet Segmentation: The study presents FuseNet segmentation which is a unique approach that utilizes deep learning techniques to achieve precise and efficient semantic segmentation. FuseNet combines multi-scale features. It also utilizes fully convolutional networks to improve the accuracy of segmentation results.

## 2 Literature review

Multi-object detection and semantic analysis in complex visual scenes have been active areas of research in the field of computer vision. In recent years, deep learning techniques have revolutionized these domains by achieving remarkable performance improvements. Various deep learning-based object detection methods have been proposed, such as Faster R-CNN (Pazhani and Vasanthanayaki, 2022) YOLO (You Only Look Once) (Diwan et al., 2023) and SSD (Single Shot MultiBox Detector) (Ahmed et al., 2021). These methods leverage convolutional neural networks (Dhillon and Verma, 2020) have challenges dealing with complex scene scenarios, occlusions, and object changes. Researchers are investigating different methods to strengthen object detection’s robustness to overcome these issues (Zhang et al., 2023), presented automated systems with the ability to accurately detect objects and determine their importance in a certain situation are essential for augmented reality, surveillance, and autonomous vehicles (Silberman et al., 2012), in their work on the NYU-Dv2 dataset, demonstrated how combining RGB and depth data can significantly improve object detection in indoor environments, addressing issues like occlusion and varying object scales. This sets the stage for our proposed approach, which aims to integrate and improve upon these methodologies.

One approach involves integrating different detection models to create a more precise system. Fusion can occur at various levels, such as feature-level fusion (Liu et al., 2017; Xue et al., 2020; Wang et al., 2020), decision-level fusion (Seong et al., 2020), or both. These fusion-based solutions aim to enhance detection accuracy and handle challenging scenarios by leveraging the strengths of multiple models. On the other hand, semantic analysis focuses on capturing high-level semantics and understanding the context of objects within an image. This research considers the categorization of objects, relationships between objects, and scene components. Unlike traditional methods relying on hand-crafted features and rule-based procedures, which have limitations in capturing complex contextual data, researchers can now utilize deep neural networks for a more comprehensive and accurate semantic analysis, thanks to advancements in deep learning.

This research proposed a unique framework called Deep Fused Networks (DFN) to support contextual scene learning. DFN provides a consistent and reliable recognition system by fusing the benefits of many object detection methods. DFN integrates several models to handle complicated scenarios like occlusions, scale variations, and crowded backgrounds. DFN also uses semantic analysis to draw out stronger semantics from visual scenes. By utilizing contextual information, the framework enables comprehension of scene attributes, object associations, and object categorization. This in-depth research enables a deeper comprehension of visual scenes and enhances decision-making.

### 2.1 Multi-object segmentation

Machine learning has been used in computer vision tasks for years, particularly in advanced applications like detecting multiple objects, recognizing scenes, and understanding contextual scenes. Numerous researchers have dedicated their efforts to exploring the visual aspects of these tasks. In Feng et al. (2020) provide a comprehensive discussion of the latest approaches and challenges in multi-modal object recognition and semantic segmentation for autonomous driving. The authors delve into the methodology, including techniques beyond deep learning, and the various datasets available for training and evaluating such systems. The paper emphasizes the complexity and challenges of these tasks within the realm of autonomous driving. In Ashiq et al. (2022) describe how developing a neural network-based object detection and tracking system can help those who are visually impaired. The authors explain how deep learning techniques are used for real-time object tracking and recognition, allowing users to intelligently navigate their surroundings. The research illustrates how this approach can improve the independence and mobility of people who are visually impaired. It offers a perceptive comprehension of how advanced technology might be applied to improve the quality of life for people with visual impairments. In Zeng et al. (2022), a new approach focused on the detection of imperfections and small-sized objects is presented by N. Zeng et al. To address the specific challenges involved in recognizing small objects, the authors suggest a multi-scale feature fusion method. They focused on the limitations of existing approaches for dealing with small objects and present an alternative that makes use of the fusion of multi-scale features to increase detection accuracy. In this paper, the authors tried to highlight the efficiency of their proposed framework by conducting experiments and an evaluation process for detecting defected objects. In Kong et al. (2022), a lightweight network model named YOLO-G is introduced by L. Kong et al. to improve the system of military target detection. They considered the challenges while improving the target detection accuracy. They presented the simplified form of the “You Only Look Once” (YOLO) technique with some modifications in accordance with the military applications. In Guo et al. (2023) the challenge of scale variation in object detection, by introducing the Multi-Level Feature Fusion Pyramid Network (MLFFPN), which effectively fuses features with different receptive fields to enhance object representations’ robustness. It utilizes convolutional kernels of varying sizes during feature extraction, reconstructs feature pyramids through top-down paths and lateral connections, and integrates bottom-up path enhancement for final predictions. In Solovyev et al. (2021) proposed based on, weighted boxes fusion, introduces a novel method for fusing predictions from various object detection models, emphasizing the utilization of confidence scores to construct averaged bounding boxes. In Cheng et al. (2023) the author addresses the challenge of accurate multi-scale object detection in remote sensing images, by getting inspiration from the YOLOX framework and proposes the Multi-Feature Fusion and Attention Network (MFANet). By reparametrizing the backbone, integrating multi-branch convolution, attention mechanisms, and optimizing the loss function, MFANet enhances feature extraction for objects of varying sizes, resulting in improved detection accuracy. In Oh and Kang (2017) accurate object detection and classification is achieved through decision-level fusion of classification outputs from independent unary classifiers, leveraging 3D point clouds and image data. The approach utilizes a convolutional neural network (CNN) with five layers for each sensor, integrating pre-trained convolutional layers to consider local to global features. By applying region of interest (ROI) pooling to object candidate regions, the method flattens color information and achieves semantic grouping for both charge-coupled device and Light Detection And Ranging (LiDAR) sensors. In Xiong et al. (2020) the author introduces a novel fusion strategy, BiSCFPN, based on a backbone network. Comprising bi-directional skip connections (BiSC), selective dilated convolution modules (SDCM), and sub-pixel convolution (SP), this strategy aims for simplicity and efficiency in high-quality object detection. BiSCFPN aims to mitigate the problems associated with traditional interpolation methods and strives to achieve a better balance between precision and speed, addressing limitations observed in current approaches.

### 2.2 Contextual scene learning

In the past, semantic segmentation for object detection and contextual scene learning has been performed manually. However, the advancement in deep learning-based image-processing techniques has improved computer vision tasks nowadays. These advanced approaches are critical to extracting complex contextual information from images, enabling more precise and efficient object detection for contextual scene-learning tasks. In Kim et al. (2020) explores the use of contextual information to improve the accuracy of monocular depth estimation. While addressing the limitations of depth estimation from a single image, the authors propose a framework that incorporates contextual cues such as object relationships and scene understanding. The framework provides detailed information over contextual information with reference to its potential for advancing monocular depth estimation techniques. In Dvornik et al. (2019) demonstrates the significance of incorporating visual context during data augmentation to enhance scene understanding models. To understand the contextual relationship between the objects they improved the robustness and generalization capabilities of the models. In Wu et al. (2020) emphasize combining pyramid pooling and transformer models to enhance the efficiency of scene understanding tasks under specific conditions. They overcame the limitations of existing methods in capturing both local and global contextual information within scenes. The authors propose P2T as a solution to effectively incorporate multi-scale features and long-range dependencies for comprehensive scene understanding. In Hung et al. (2020) the authors introduce the Contextual Translation Embedding approach, which incorporates contextual translation to improve the accuracy and contextual understanding of visual relationships. They contribute to the field of detecting visual relationships and scene graph generation. Additionally, they offer possible developments in capturing fine-grained details and spatial patterns within visual scenes. In Chowdhury et al. (2023) a unique approach is introduced by extending the representation to encompass human sketches, creating a comprehensive trilogy of scene representation from sketches, photos, and text. Unlike rigid three-way embedding, the focus is on a flexible joint embedding that facilitates optionality across modalities and tasks, allowing for versatile use in downstream tasks such as retrieval and captioning. Leveraging information-bottleneck and conditional invertible neural networks, the proposed method disentangles modality-specific components and synergizes modality-agnostic instances through a modified cross-attention mechanism, showcasing a novel and flexible approach to multi-modal scene representation. In Hassan et al. (2020) novel approach is introduced by integrating handcrafted features with deep features through a learning-based fusion process, aiming to enhance detection accuracy under challenging conditions such as intraclass variations and occlusion. This work builds upon the YOLO object detection architecture, aligning with the contemporary trend of leveraging deep learning methods for improved object localization and recognition in complex real-life scenarios.

The summary of the above studies has been incorporated with all the required fields in a table to clearly elaborate the findings and limitations of the existing studies as follows:

**Table:** 

References	Methods	Evaluation metrics	Limitations
Pazhani and Vasanthanayaki (2022)	Faster R-CNN	Accuracy, mAP	Challenges with complex scenes, occlusions, and object changes
Diwan et al. (2023)	YOLO (You Only Look Once)	Accuracy, FPS	Low accuracy on cluttered scenes understanding
Ahmed et al. (2021)	SSD (Single Shot MultiBox Detector)	Accuracy, mAP	Occlusion detection, complex multi-object detection
Dhillon and Verma (2020)	Convolutional Neural Networks	Accuracy	Scenes with occluded objects and time complexity
Zhang et al. (2023)	Automated object detection systems	Accuracy, Robustness	Handling complex scenarios
Silberman et al. (2012)	NYU-Dv2	Accuracy, Precision	Focused on indoor environments only
Liu et al. (2017), Xue et al. (2020), Wang et al. (2020)	Feature-level fusion	Detection Accuracy	Fusion and time complexity
Seong et al. (2020)	Decision-level fusion	Detection Accuracy	Fusion complexity with varied environmental challenges
Feng et al. (2020)	Multi-modal object recognition and semantic segmentation KITTI	Accuracy, mAP	Complexity and challenges in autonomous driving scenarios
Ashiq et al. (2022)	Neural network-based object detection and tracking	Real-time performance, Accuracy	Real-time constraints, application for visually impaired
Zeng et al. (2022)	Multi-scale feature fusion	Detection Accuracy	Recognition of small objects
Kong et al. (2022)	YOLO-G (lightweight YOLO)	Detection Accuracy, Speed	Adaptation for military applications
Guo et al. (2023)	Multi-Level Feature Fusion Pyramid Network (MLFFPN)	Detection Accuracy, Robustness	Scale variation handling
Solovyev et al. (2021)	Weighted boxes fusion	Bounding Box Accuracy	Fusion complexity
Cheng et al. (2023)	Multi-Feature Fusion and Attention Network (MFANet)	Detection Accuracy	Accurate multi-scale object detection in remote sensing images
Oh and Kang (2017)	Decision-level fusion using 3D point clouds and image data	Accuracy, Robustness	Integration complexity
Xiong et al. (2020)	BiSCFPN (bi-directional skip connections, selective dilated convolution)	Detection Accuracy, Speed	The balance between precision and speed
Kim et al. (2020)	Contextual cues for monocular depth estimation	Depth Estimation Accuracy	Single image limitations
Dvornik et al. (2019)	Visual context data augmentation	Scene Understanding Accuracy	Robustness and generalization challenges
Wu et al. (2020)	Pyramid pooling and transformer models	Scene Understanding Efficiency	Capturing local and global contextual information
Hung et al. (2020)	Contextual Translation Embedding	Visual Relationship Accuracy	Fine-grained detail and spatial pattern limitations
Chowdhury et al. (2023)	Flexible joint embedding for multi-modal scene representation	Retrieval and Captioning Performance	Complexity in modality-specific and modality-agnostic components
Hassan et al. (2020)	Handcrafted and deep feature fusion	Detection Accuracy	Challenges with intra-class variations and occlusion
He et al. (2023)	Adaptive Self-supervised Transformer (AST) utilizing Masked Image Modeling, cross-scale Transformer architecture, & adaptive masking token strategy.	Object Detection Accuracy	High computational cost due to Transformer architecture, Complexity in implementing adaptive masking strategy, Potential difficulty in scaling for extremely large datasets
He (2024)	Grouping Prompt Tuning Framework (GoPT), including class-aware uni-modal prompter and alignment-induced cross-modal prompter	Pixel Accuracy, mIoU, Mean Accuracy	Limited by the frozen pre-trained foundation model, Potential issues with scalability for more complex tasks, Dependency on effective semantic grouping for optimal results
Wang et al. (2023)	Calibration-guided source-free domain adaptive semantic segmentation (Cal-SFDA framework)	Mean Intersection over Union (mIoU)	- Potential complexity in implementing the LogSumExp trick and value net for ECE estimation. May require extensive computation for model training and ECE estimation

## 3 Materials and methods

### 3.1 System methodology

**Figure 1:**
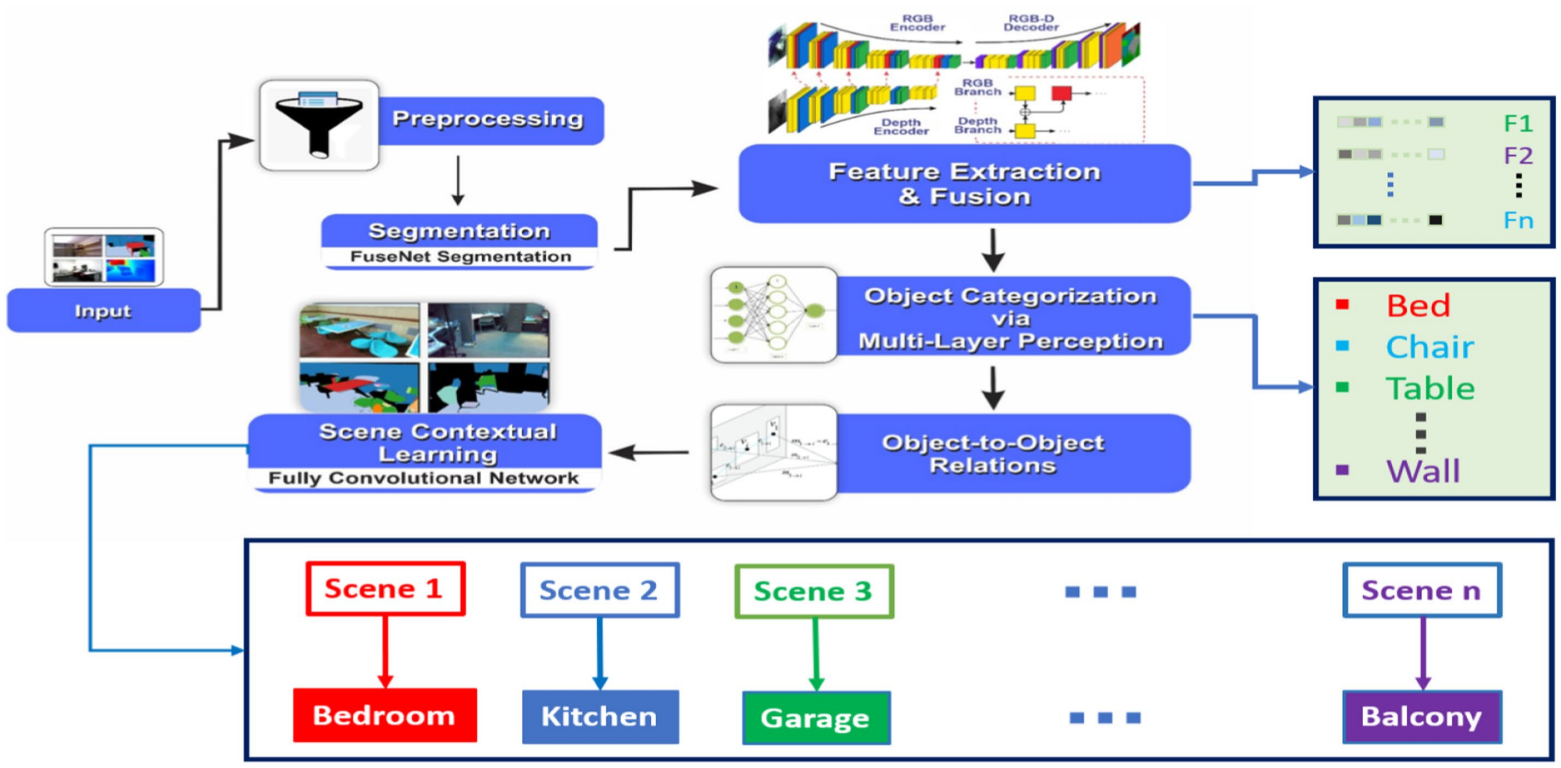
Schematic view of the proposed model for contextual scene learning.

In this section, we present our methodology for contextual scene learning using a multi-stage approach. The process is comprised of multiple steps. Initially, we start with the input acquisition, followed by preprocessing to enhance the quality and consistency of the data. We then employ the FuseNet segmentation network to extract pixel-wise semantic information from the input images. Next, feature extraction and fusion through various techniques from multiple modalities, comprehensive information is acquired to proceed with object categorization. Subsequently, to assign the semantic labels to each individual object within the scene, object categorization is incorporated. Once the objects are classified into various categories, object-to-object relationship modeling is then employed to gather the contextual information of these objects. Finally, a fully convolutional network is employed for contextual scene learning, enabling a holistic understanding of the scene and its semantic context as shown in Figure 1.

### 3.2 Pre-processing

In the context of RGB and depth images used for scene understanding, noise is commonly observed during pre-processing, particularly in regions with low texture or reflective surfaces. To minimize the effects of noise on further analysis, noise reduction techniques are employed. Among these techniques, Gaussian or bilateral filtering methods (Zhang and Gunturk, 2008) are frequently applied to depth images. These techniques are effective in effectively reducing the presence of noise and smoothing images while preserving essential structural information. Mathematically, we can express the bilateral filter as follows (see Equation 1):


(1) $$\begin{array}{l} B\left(x,y\right)=\left(\frac{1}{W\left(x,y\right)}\right)\;\underset{i,j\in \;\Omega \left(x,y\right)}{\sum }I\left(i,j\right)\;G_{Spatial}\left(\left||\left(x,y\right)-\left(i-j\right)|\right|\right)\;\\ \;\;\;\;\;\;\;\;\;\;\;\;\;\;\;\;\;\;\;\;\;G_{Intensity}\left(\left|I\left(x,y\right)-I\left(i,j\right)\right|\right) \end{array}$$

where (x,y) denotes the coordinates of the pixel being filtered, (i,j) means the coordinates of the neighboring pixel, Ω(x,y) means neighborhood pixels around pixel (x,y) , I(x,y) is the intensity value of the neighboring pixel, $G_{Spatial}$ is the spatial Gaussian kernel that captures the spatial proximity between pixels, $G_{Intensity}$ is the intensity Gaussian kernel that measures the similarity of pixel intensities, W(x,y) is the normalization factor that ensures the sum of weights is equal to 1 and can be expressed as (Equation 2):


(2) $$\begin{array}{l} W\left(x,y\right)=\underset{i,j\in \;\Omega \left(x,y\right)}{\sum }G_{Spatial}\left(\left||\left(x,y\right)-\left(i-j\right)|\right|\right)\;\\ \;\;\;\;\;\;\;\;\;\;\;\;\;\;\;\;\;\;\;\;\;\;\;G_{Intensity}\left(\left|I\left(x,y\right)-I\left(i,j\right)\right|\right) \end{array}$$

The bilateral filter is effective in different aspects when compared with the Gaussian filter. Specifically, it is superior in terms of preserving edge information while removing noise from the input image. The bilateral filter considers both the spatial proximity and pixel intensity differences during the noise removal or smoothing process.

### 3.3 FuseNet segmentation

**Figure 2:**
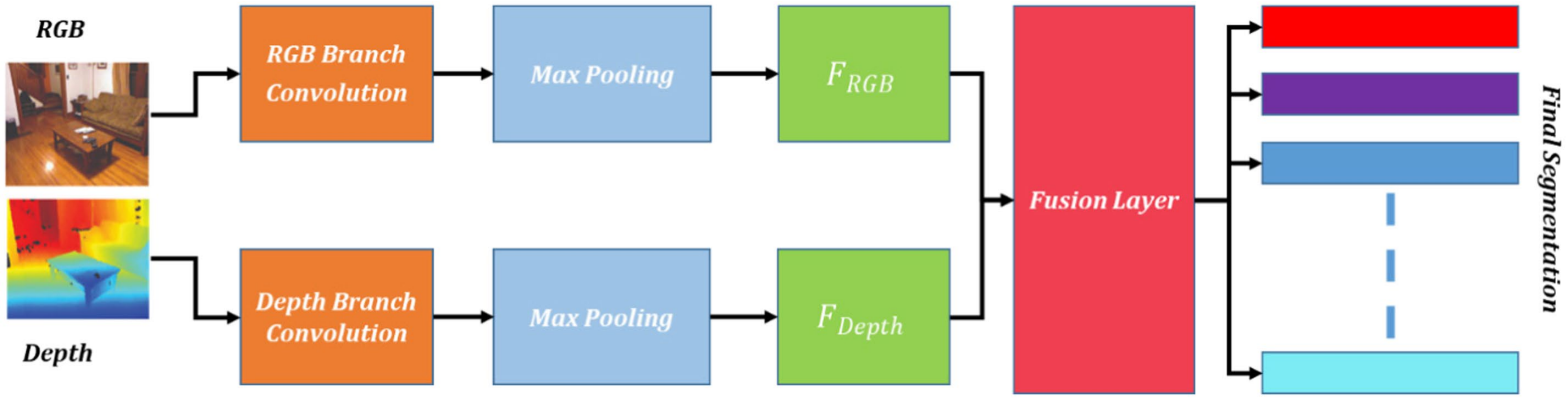
Schematic view of FuseNet segmentation.

The FuseNet method, designed for semantic segmentation and understanding contextual scenes which combines RGB and depth information to label each pixel in a scene accurately. Its goal is to capture features based on both appearances from RGB images and geometric aspects from depth information, enhancing the accuracy of segmentation. The FuseNet architecture typically involves two branches: the RGB branch and the depth branch. Each branch processes the respective input modality and extracts relevant features. The output feature maps from both branches are then fused together to generate the final segmentation result as shown in Figure 2 using Equations 3, 4.


(3) $$F_{RGB}=MaxPool\;\left(I_{RGB},K_{size}=3,St=2\right)$$

(4) $$F_{Depth}=MaxPool\;\left(I_{Depth},K_{size}=3,\;St=2\right)$$

where $F_{RGB}$ denotes the RGB features while $F_{Depth}$ means the depth features, extracted by RGB and depth branches of FuseNet Segmentation architecture, respectively. $F_{RGB}$ and $F_{Depth}$ represent the downsampling of features obtained from the RGB and depth branches, achieved through the MaxPool operation. The MaxPool operation involves traversing the input features with a 3×3 kernel, selecting the maximum value within each region, and moving with a stride of 2, indicating the number of pixels the kernel shifts during each step. These operations result in down-sampled feature maps that capture essential information while reducing spatial dimensions. The fusion of the feature maps may be expressed mathematically as follows using (Equation 5):


(5) $$F_{Fused}=W_{RGB}*F_{RGB}+W_{Depth}*F_{Depth}$$

where $W_{RGB}$ is the weight assigned to RGB images, while $W_{Depth}$ represents the weights assigned to depth images. Similarly, * denotes the element-wise multiplication for fusion, and + denotes the element-wise addition. The general FuseNet process can be represented mathematically as follows (see Equation 6):


(6) $$S=FuseNet\left(I_{RGB},I_{Depth}\right)$$

**Figure 3:**
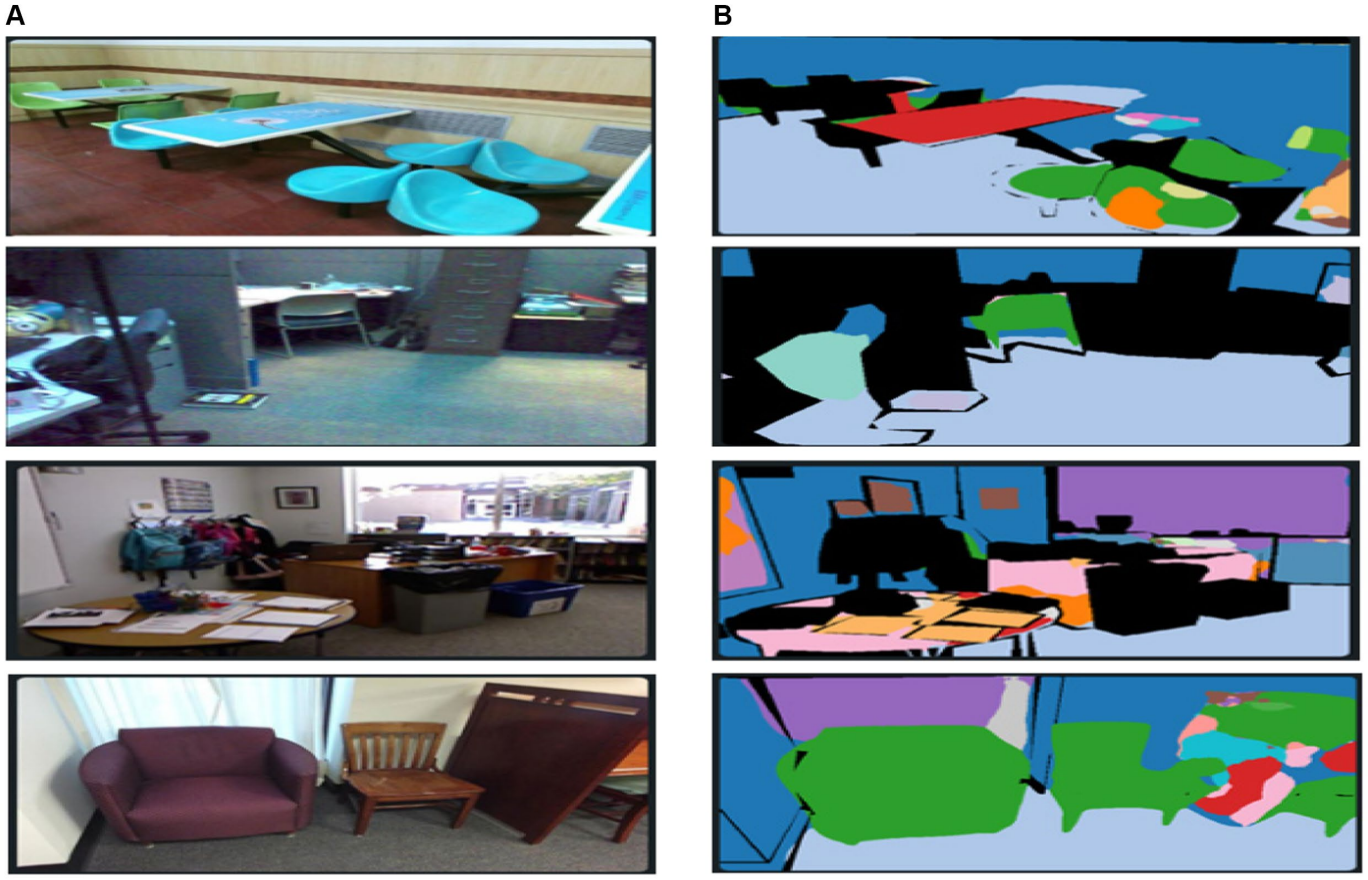
Results of FuseNet segmentation over some images from the SUN-RGB-D dataset. **(A)** Original images and **(B)** segmented images.

A commonly used initial learning rate is 0.01. However, with the passage of time and an increasing number of epochs that reduce the learning rate to adaptively adjust the learning rate during training based on the model’s performance. FuseNet consists of 6 convolutional layers interleaved with pooling layers. However, the depth of the network can be adjusted based on the complexity of the RGB-D datasets and the available computing resources. Moreover, we use 3×3 filters in the convolutional layers to capture the local context. Strides of 2×2 are used in pooling layers for down-sampling and spatial resolution reduction. For semantic segmentation of RGB-D datasets, the combination of cross-entropy loss and Dice loss is used as described in Equations 7, 8, respectively.


(7) $$L_{CE}=-\overset{n}{\underset{i=1}{\sum }}y_{i}\;\log\left(p_{i}\right)$$

(8) $$L_{DICE}=1-2*\overset{n}{\underset{i=1}{\sum }}\left(y_{i}\;p_{i}\right)/\left(y_{i}+p_{i}\right)$$

where $y_{i}$ denotes the ground truth label while $p_{i}$ represents the predicted probability for pixel i, respectively. The cross-entropy loss helps to optimize the pixel-wise class predictions, while the Dice loss encourages better overlap between the predicted and ground truth segmentation masks. The relative weight between these losses can be adjusted based on the dataset characteristics. The results of FuseNet segmentation are demonstrated in Figure 3.

### 3.4 Feature extraction

To extract the deep features from segmented objects are taken as input and can be expressed as follows segmentation. We can identify regions with similar color or texture characteristics by grouping similar pixels into clusters, which can then be used as inputs for region-based segmentation (see Equation 9).


(9) SObj∈ℝ^(H×W×C)

where H and W denote the height and width of the segmented image, respectively. While C represents the number of channels. Each pixel of the segmented object can also be represented as $S_{Obj}\;\left(i,j,k\right)$ where i,j are the coordinates of the segmented image, and k denotes the index of the channel. The input is processed for convolution over the convolution layer where filters (kernels) are used. These filters are denoted by weight matrix $W_{m}$ having a size $F\times F\times C_{Pre}$ where F represents the size of the filter, and $C_{Pre}$ is the number of channels from the previous layer. The convolution process is computed mathematically as follows (Equation 10):


(10) $$F_{m\_con}=A_{f}\left(W_{m},S_{Obj}+b\right)$$

where $A_{f}$ represents the activation function, $F_{m}$ denotes the output feature map of the convolutional layer, $W_{m}$ is the weight matrix, $\;S_{Obj}$ is the input of the layer, which is segmented objects, and b denotes the bias. The output feature map is forwarded to the pooling layer to reduce its dimensions by converting the overlapping regions into non-overlapping regions. The size of regions is defined by P×P . After the dimensions are reduced from the pooling layer, the feature map has the following dimensions (see Equation 11):


(11) $$\left(W_{pool},H_{pool}+C_{out}\right)$$

where $H_{pool}$ is the height, $W_{pool}$ is the width of the output feature map of the pooling layer and $C_{out}$ is to denote the number of channels. The feature maps obtained from convolutional and pooling layers are flattened into a single dimension as a vector to serve as input to the fully connected layer.

**Figure 4:**
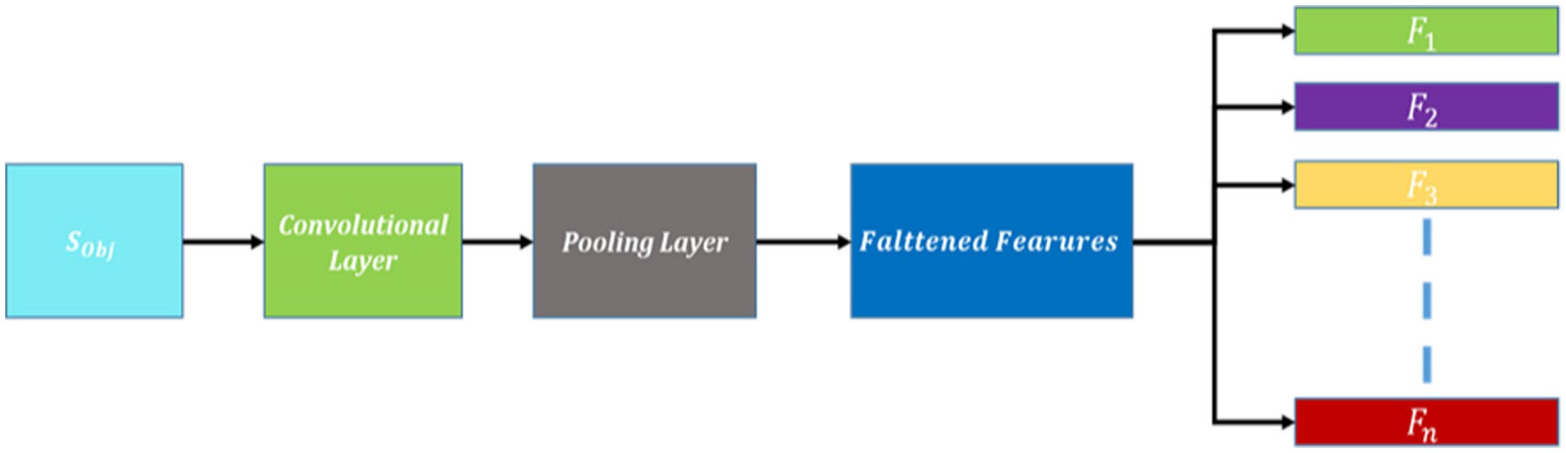
Schematic diagram of feature extraction using CNN.

The decoder is used to reconstruct the encoded feature maps to the original resolution of the input image. This process involves transposed convolutions (deconvolutions) to up-sample the feature maps. The goal of the decoder is to generate high-resolution feature maps that accurately represent the spatial context and details of the original segmented objects. The details are as follows:

The encoder outputs a feature map of size 16 × 16 × 128 (height, width, channels). Apply a transposed convolution with a filter size of 3 × 3, a stride of 2, and appropriate padding. This operation up-samples the feature map to 32 × 32 × 64. Then another transposed convolution with similar parameters is applied that up-sample the feature map to 64 × 64 × 32. Finally, a transposed convolution to match the original input image resolution is applied that will up-sample the feature map to 128 × 128 × 3. The schematic view of feature extraction using CNN is demonstrated in Figure 4.

### 3.5 Object categorization via MLP

**Figure 5:**
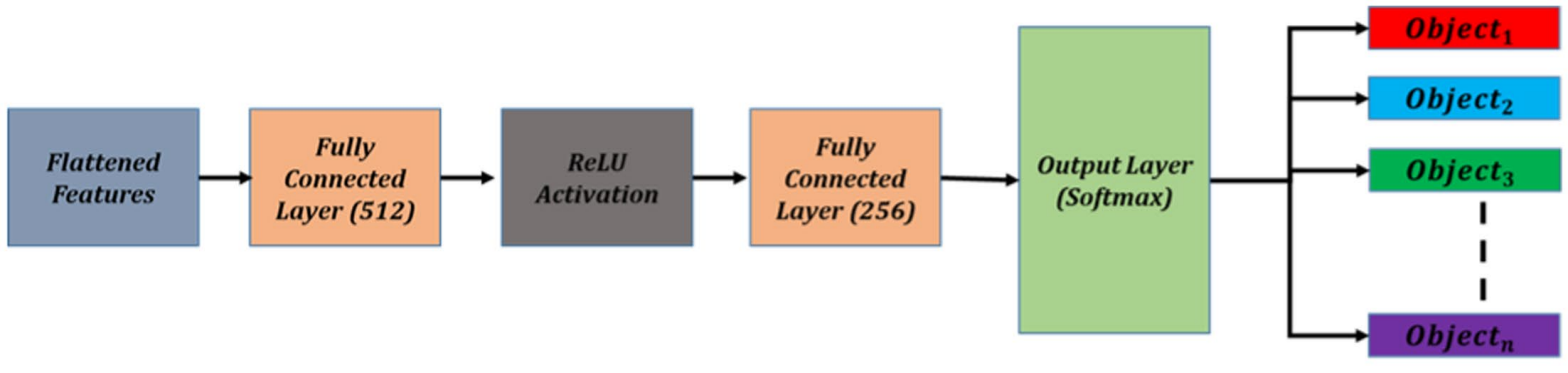
Schematic diagram of object categorization using MLP.

For the MLP architecture, we designed a two-layer fully connected network. The first hidden layer consists of 512 neurons, followed by a ReLU activation function to introduce non-linearity. The second hidden layer consists of 256 neurons, also followed by a ReLU activation function. The output layer contains as many neurons as the number of object categories in the SUN RGB-D dataset, with a softmax activation function to generate class probabilities. Figure 5 illustrate the details of object categorization.

**Figure 6:**
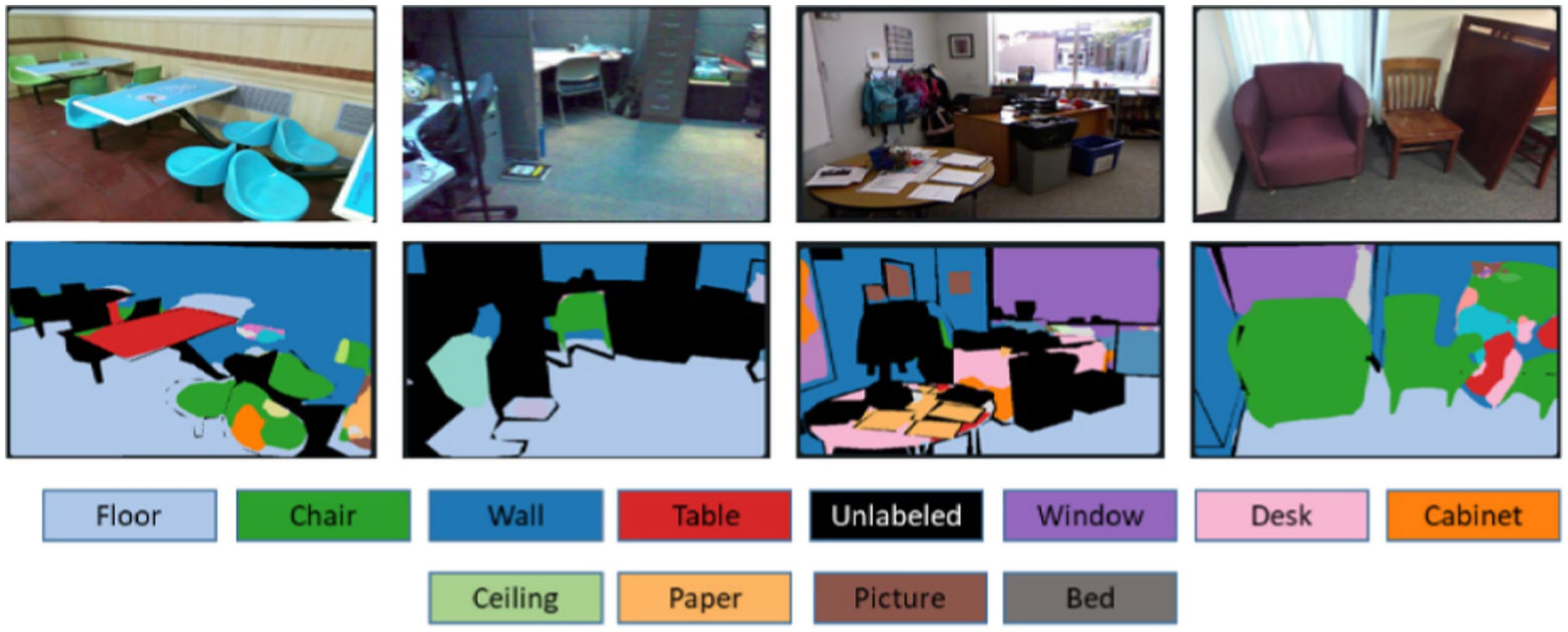
Object categorization results by applying MLP over the SUN RGB-D dataset.

The neurons in the layers of MLP can be expressed as by using Equation 12 as follows:


(12) $$y_{ij}=f\left(\sum \left(W_{ij}*X_{ij}+b_{ij}\right)\right)$$

where $y_{ij}$ is the output, $X_{ij}$ is the input of the ith neuron at layer j , while $W_{ij}$ denotes the weights associated with these inputs, $b_{ij}$ is to represent bias and f is the activation function applied to the weighted sum. To update the weights $W_{ij}$ (Equation 13), the following backpropagation is used:


(13) $$\Delta W_{ij}=-\eta *\partial E/\partial W_{ij.}$$

where $\Delta W_{ij}$ the rate of change in weights is, η is the learning rate, E is the total error and can be written as $E=L_{CE}+L_{DICE}$ , and $\partial E/\partial W_{ij}$ denotes the partial derivative of the error with respect to the weight $\;W_{ij}$ . The object categorization results are presented in Figure 6.

### 3.6 Object–to–object (OOR) relationship

Graph-based object-to-object relationships (Hassan et al., 2020) provide a powerful setting for contextual scene learning, enabling a structured representation of the relationships and interactions between objects within a scene. To understand a scene comprehensively, objects are modeled as nodes having attributes such as their semantic label, position, and size while their relationships are considered as edges. These relationships can be classified into different types, such as containment, proximity, support, or interaction during scene understanding tasks or contextual scene learning.

To construct the object-to-object relationship graph, a technique to analyze the spatial attributes of categorized objects Obj , considering factors such as distance, overlap, or relative positions is applied. Let us consider OOR graph as general graph G=VE where V means the set of nodes or detected objects Obj while E denotes the set of edges or relationships between these categorized objects, and G is equivalent to OOR which is the relationship between these objects. The relationship between objects can be expressed as an adjacency matrix as described in Equation 14 below.


(14) A∈{0,1}^(|Obj|×|Obj|)

where A=1 if there is a relationship between the objects and A=0 otherwise.

Let $y_{I}$ be the scene labels, and OOR be the set of possible semantic relationships between objects. The mathematical function of OOR can be represented as follows (see Equation 15):


(15) $$OOR=f:Obj\to y_{I}$$

The contextual scene learning system utilizes the capabilities of graph-based object-to-object relationships to accomplish holistic scene understanding. This mechanism supports higher-level reasoning, object interaction analysis, and contextual inference. Moreover, other vision tasks such as multi-object detection, scene understanding, and contextual scene learning can be expanded and revolutionized.

### 3.7 Contextual scene learning via FCN

**Figure 7:**
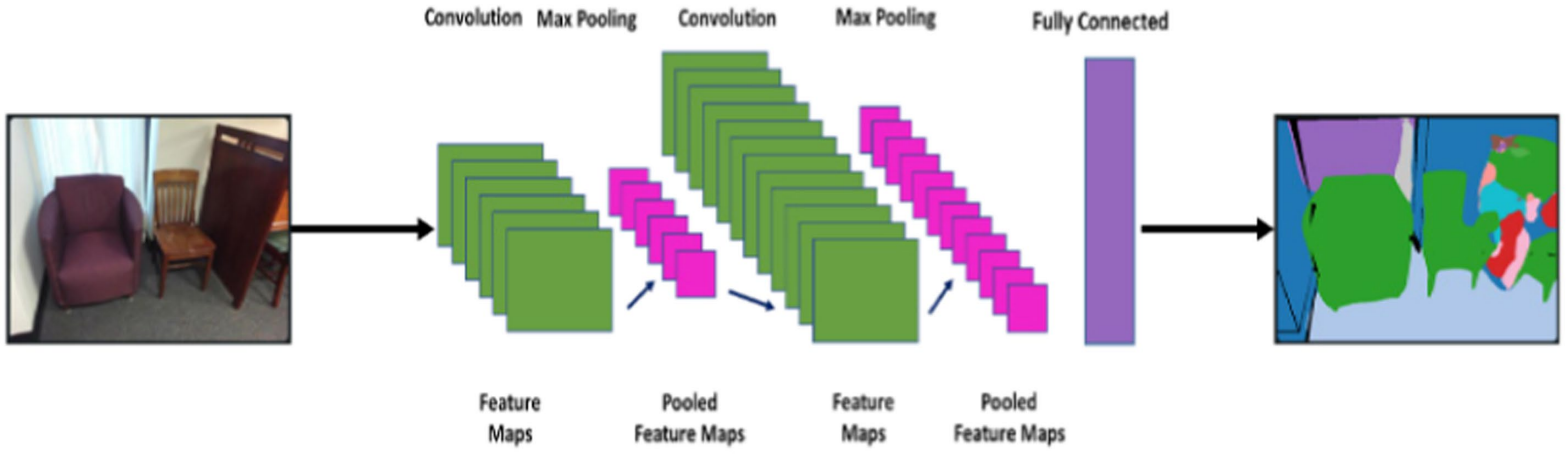
Object categorization results by applying MLP over the SUN RGB-D dataset.

FCN is one of the most used deep learning models from Convolutional Neural Networks that is used to perform multiple tasks including object categorization, semantic segmentation, scene recognition, and classification. There are numerous advantages of the model, however, the fundamental benefit of FCN over other traditional CNNs is its capability to take the input image without resizing constraints and process it accordingly. The FCN architecture is illustrated in Figure 7.

Let us consider in input image (objects) as $\;x_{I}$ , and the predicted label of the scene as $y_{I}$ . Initially, the FCN is supposed to take $x_{I}$ and the OOR as input and predict scene label $\;y_{I}$ . The FCN comprised multiple convolutional layers, each of which applies a convolutional filter to the input image. Convolutional filters are learned during training to extract features that are relevant to the task of scene recognition. The OOR relations are a set of pairwise relationships between objects in the scene as computed in the previous section. The OOR relations are used to add additional information to the feature map. Here, is a more mathematical representation of the FCN architecture (see Equation 16):


(16) $$FCN\left(x_{I},OOR\right)=argmax_{y}\;p(y_{I}\;|\;x_{I},OOR)$$

where $x_{I}$ denotes the input image features, OOR means object-to-object relations, $y_{I}$ represents the scene label for scene image I, while $p(y_{I}\;|\;x_{I},OOR)$ means the probability of the particular scene label $y_{I}$ when given the input image features $x_{I}$ . The complete flow of the FCN is described in the Algorithm 1.

**Table:** 

**Algorithm 1INPUT**: $x_{I},OOR$ ; images (objects), Object-to-Object Relations **OUTPUT**: scene label 1. Normalize the input image (object) $\;x_{I}$ . 2. Initialize the FCN with multiple convolutional layers 3. Set up convolutional filters and activation functions 4. For each convolutional layer: Apply convolution, activation function, and pooling to $\;x_{I}$ . 5. Integrate OOR data into $\;x_{I}$ . 6. For each transposed convolutional layer: Apply transposed convolution to $\;x_{I}$ . 7. Apply the softmax function to $x_{I}$ to get the scene label $\;y_{I}$ . 8. Compute the loss between $y_{I}$ and the ground truth. 9. Perform backpropagation to update the model weights. 10. Train the FCN model using the dataset. 11. Evaluate the trained FCN model on the validation dataset. 12. Use the trained FCN model to predict the scene label $y_{I}$ for new input images $\;x_{I}$ . RETURN: The predicted scene label $y_{I}$ .

## 4 Experiments and results

This section delves into the dataset particulars and the intricacies of the research, covering aspects like the experimental configuration, the effectiveness of the proposed system, and a comparative analysis with state-of-the-art techniques.

### 4.1 Datasets

For the purposes of our study, we used three complex datasets, including SUN RGB-D, NYU-Dv2, and SYNTHIA datasets. The details of these datasets are given as follows.

#### 4.1.1 SUN RGB-D dataset

**Figure 8:**
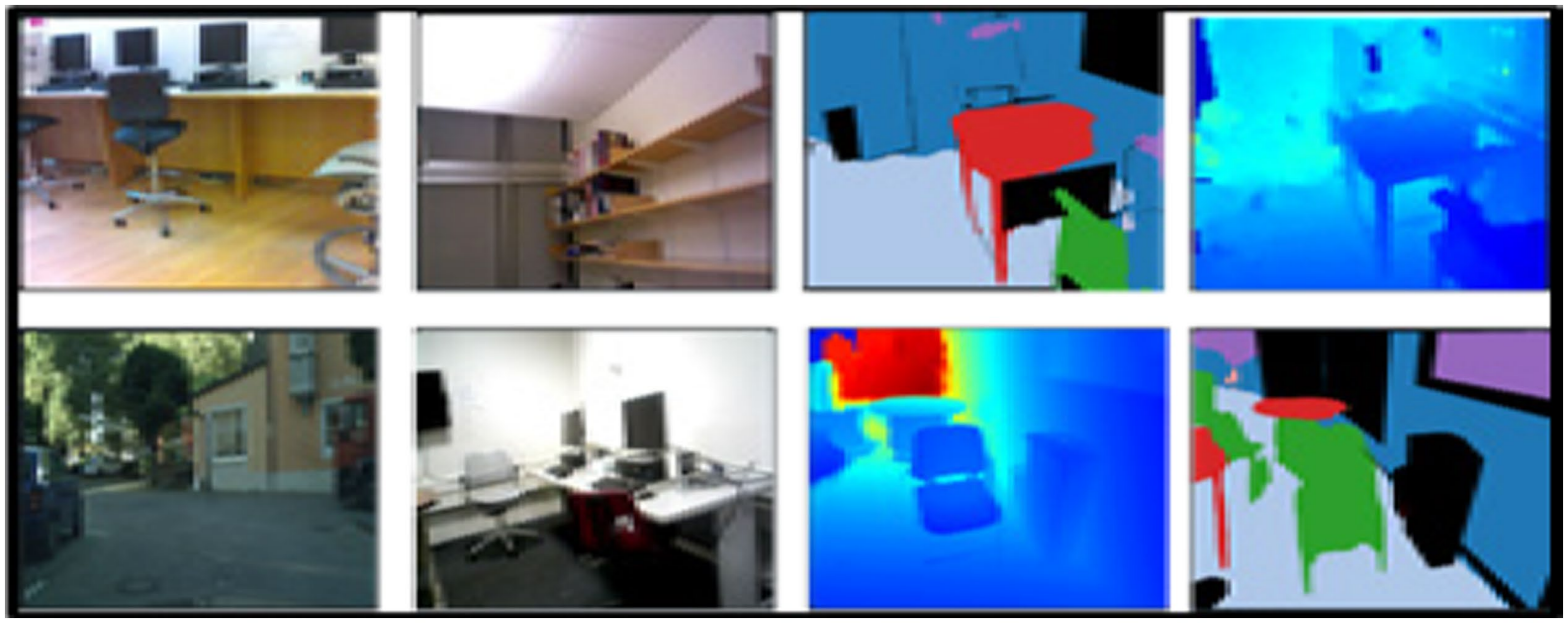
A few examples from the SUN RGB-D dataset.

The SUN RGB-D dataset (Song et al., 2015) with 10,355 RGB-D images is a complex dataset. The dataset has 19 categories comprising multiple images in each category of SUN RGB-D. It is a collection of NYU-Dv2, Berkeley B3DO, and SUN3D RGB-D image datasets. The two parts of the dataset: training and testing have 5,510 and 4,845 images, respectively Figure 8 depicts a few examples of the SUN RGB-D dataset.

#### 4.1.2 NYUDv2 dataset

**Figure 9:**
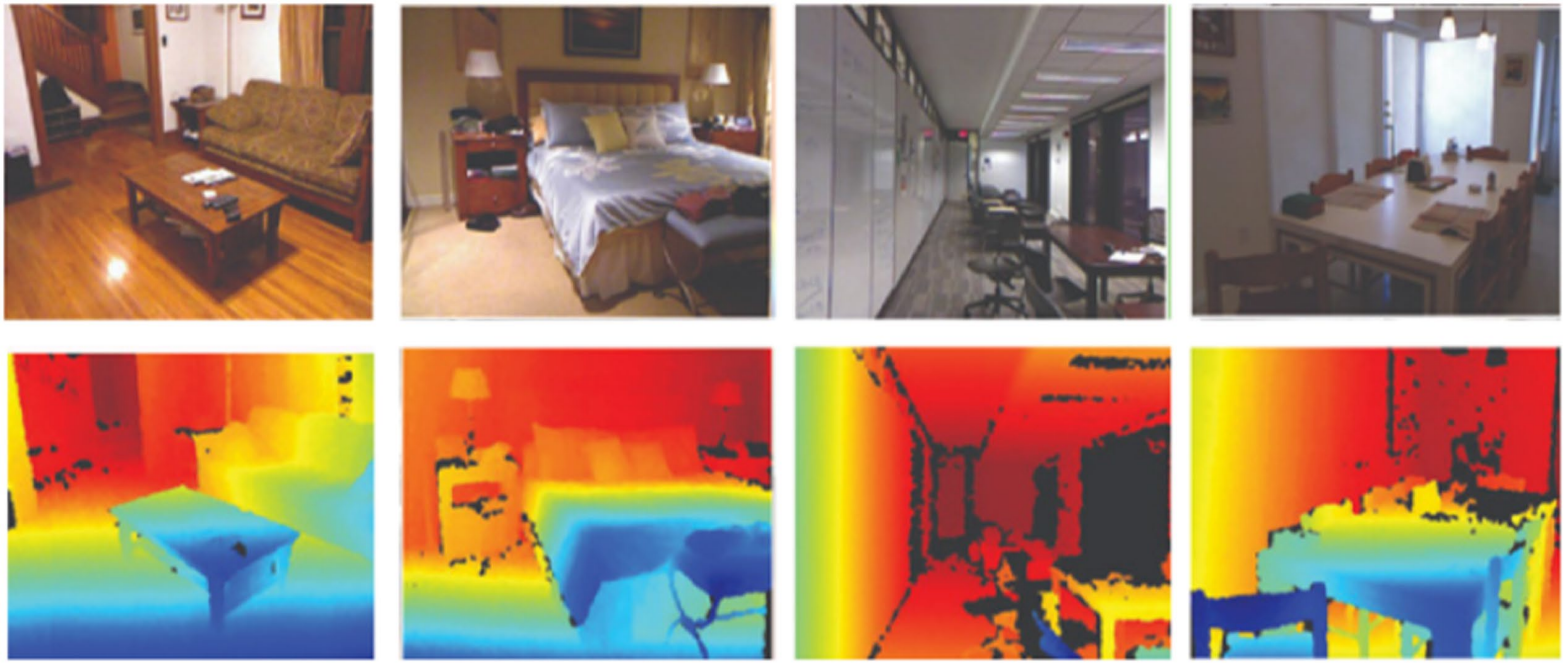
A few examples from the NYU-Dv2 dataset.

The NYUDv2 dataset (Silberman et al., 2012) consists of labeled and unlabeled frames of various scenes. There are 2,347 labeled and 108,617 unlabeled frames having one of the 7 categories with 64 different indoor scenarios. These scenes may be categorized into one of the seven classes including bathroom, bedroom, bookstore, café, kitchen, living room, and office. Each class has some objects like a bed, bookshelf, background, unlabeled, etc. Figure 9 illustrates some images from the NYU-Dv2 dataset.

#### 4.1.3 SYNTHIA dataset

**Figure 10:**
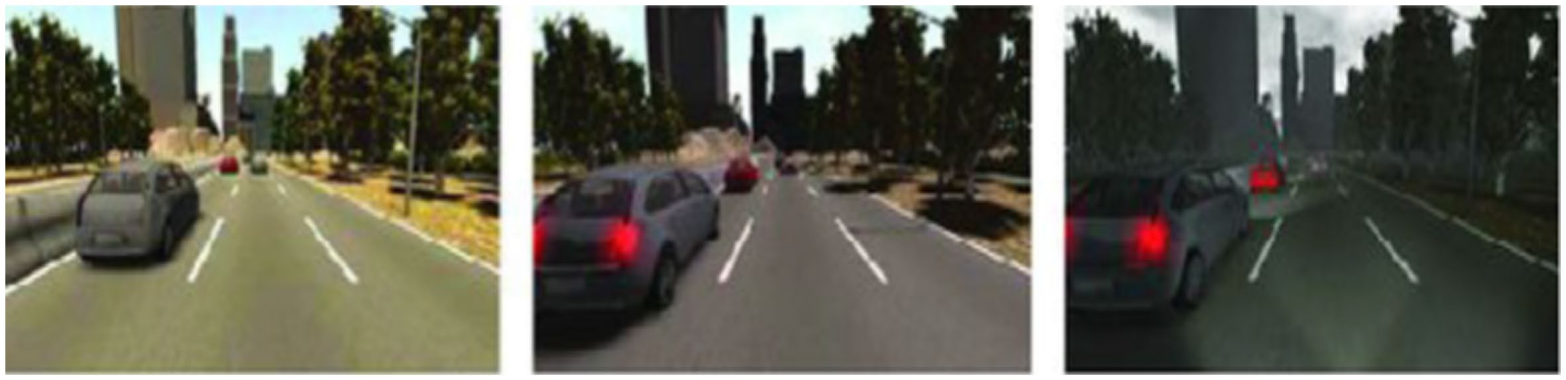
A few examples from the SYNTHIA dataset.

The SYNTHIA dataset (Ros et al., 2016) comprised 9,400 having 13 classes of synthetic images. These images are synthesized from a virtual city. The resolution of all the images/frames in the dataset is 1,280 × 960. Initially, a video stream is generated at 25fps and then converted to a sequence of frames. The dataset includes the following categories: car, fence, void, sidewalk, traffic-sign, bicycle, lane-marking, traffic-light, etc. The dataset contains all the necessary information including semantic segmentation labels, 2D and 3D bounding boxes along with the depth of the images. A few example images of the SYNTHIA dataset are shown in Figure 10.

### 4.2 Experimentations and results

In this section, we conducted a set of experiments to evaluate the proposed model’s detection and classification accuracy across benchmark datasets. The objective was to validate its efficacy in comparison to other established methods.

#### 4.2.1 Quantitative analysis

To evaluate the presented model, three benchmark datasets, namely SUN RGB-D, NYU-Dv2, and SYNTHIA are used. The performance evaluation of the proposed model involved computing various metrics such as mean accuracy, sensitivity as Equation 17, true negative rate (TNR) as Equation 18, and F1 Score as Equation 19. The confusion matrices of recognition accuracies over SUN RGB-D and NYU-Dv2 datasets are presented in Tables 1, 2, respectively. The high recognition accuracy across classes, with a mean accuracy of 94.27%, underscores the robustness of the proposed method in diverse indoor scenes. The confusion matrix showcases minimal misclassifications, emphasizing the model’s ability to distinguish between objects such as chairs, tables, sofas, and walls. Despite the challenges posed by different furniture and scene contextual classes in NYU-Dv2 datasets, the proposed method achieves a mean accuracy of 72.8%. Notably, the confusion matrix reveals successful classification across various classes, demonstrating the model’s capability to discern between objects like beds, bookshelves, and televisions. Detailed analysis of the evaluation metrics can be found in Table 3 for the SYNTHIA dataset which simulates diverse outdoor scenarios and showcases a remarkable mean accuracy of 95.08%. The confusion matrix highlights the model’s proficiency in distinguishing between outdoor elements like sky, buildings, roads, and pedestrians. To ensure fairness, a separate set of unseen samples from the test data was used for evaluation. The results demonstrated outstanding performance, surpassing existing state-of-the-art techniques.

**Table 1:** Confusion matrix for recognition accuracy over SUN-RGBD dataset.

Class	CH	CT	SO	TA	BO	CA	CB	CM	SC	WL	FR
CH	0.99	0	0.01	0	0	0	0	0	0	0	0
CT	0	0.89	0	0	0	0	0.11	0	0	0	0
SO	0.03	0	0.97	0	0	0	0	0	0	0	0
TA	0	0	0.02	0.98	0	0	0	0	0	0	0
BO	0	0	0	0.01	0.99	0	0	0	0	0	0
CA	0	0	0	0	0	0.89	0	0.02	0	0	0
CB	0	0	0	0	0.13	0	0.87	0	0	0	0
CM	0	0	0	0	0	0	0	0.93	0	0.07	0
SC	0	0	0	0	0	0	0	0.03	0.97	0	0
WL	0	0	0	0	0	0	0	0	0	0.98	0.02
FR	0	0.02	0	0.11	0	0	0	0	0	0.12	0.91
**Mean accuracy = 94.27%**											

CH, chair; CT, coffee table; SO, sofa; TA, table; BO, bowl; CA, cap; CB, cereal box; CM, coffee mug; SC, soda can; WL, wall; FR, floor.

**Table 2:** Confusion matrix for scene contextual classification accuracy over NYU-Dv2 dataset.

Class	BD	BK	CB	CL	FR	SF	TB	TV	WL	WN
BD	0.75	0	0	0	0.14	0	0.11	0	0.75	0
BK	0	0.79	0.07	0	0	0	0	0.14	0	0.79
CB	0.05	0	0.69	0	0.12	0	0	0	0.05	0
CL	0	0	0	0.77	0	0	0	0	0	0
FR	0.05	0	0	0	0.76	0	0.13	0	0.05	0
SF	0	0	0.12	0	0	0.69	0.12	0	0	0
TB	0.24	0	0.03	0	0	0	0.73	0	0.24	0
TV	0	0.25	0	0	0	0	0	0.75	0	0.25
WL	0	0	0	0.17	0	0	0	0	0	0
WN	0	0.13	0	0	0	0	0	0	0	0.13
**Mean accuracy = 72.8%**										

BD, Bed; BK, Book; CB, Cabinet; CL, Ceiling; FR, Floor; SF, Sofa; TB, Table; TV, Television; WL, Wall; WN, Window.

**Table 3:** Confusion matrix for scene contextual classification accuracy over the SYNTHIA dataset.

Class	SK	BD	RD	SW	FN	VG	PL	CR	SN	PD	CT
SK	0.98	0	0	0	0	0	0.02	0	0	0	0
BD	0	0.95	0.05	0	0	0	0	0	0	0	0
RD	0.04	0	0.96	0	0	0	0	0	0	0	0
SW	0	0	0	0.95	0	0	0	0	0.05	0	0
FN	0.05	0	0	0	0.95	0	0	0	0	0	0
VG	0	0	0	0	0	0.94	0.06	0	0	0	0
PL	0	0	0	0	0	0	0.92	0	0	0.08	0
CR	0	0	0	0	0	0	0	0.97	0	0	0
SN	0	0	0	0	0	0	0	0	0.98	0	0.02
PD	0	0	0	0.06	0	0	0	0	0	0.94	0
CT	0	0.05	0	0	0.03	0	0	0	0	0	0.92
Mean accuracy = 95.08%											

SK, Sky; BD, Building; RD, Road; SW, Sidewalk; FN, Fence; VG, Vegetate; PL, Pole; CR, Car; SN, Sign; PD, Pedestrian; CT, Cyclist.

Table 4 provides a detailed evaluation of vehicle detection results over the SYNTHIA dataset, including recognition accuracy, true positive rate, sensitivity, F1 score, specificity, and average computational time. The proposed method achieves high recognition accuracy across multiple classes which demonstrates its effectiveness in identifying various objects within the scenes. The true positive rates, sensitivity, and F1 scores also highlight the model’s ability to accurately detect and classify objects, emphasizing its precision and reliability.

**Table 4:** The overall accuracy, precision, recall, F1 score, specificity, and computational time for vehicle detection results were obtained using customized pyramid pooling over the SYNTHIA dataset.

Class	Recg. Acc. %	TPR %	Sensitivity %	F1 score	Specificity %	ACT in Sec
SK	0.9727	0.9646	0.9433	0.9538	0.923	181
BD	0.9666	0.9658	0.8719	0.9165	0.905	201
RD	0.9801	0.9602	0.8673	0.9114	0.911	185
SF	0.9475	0.9588	0.9789	0.9687	0.901	217
VG	0.9857	0.9643	0.8529	0.9052	0.856	213
PL	0.9129	0.9418	0.8845	0.9123	0.891	225
MK	0.9389	0.8955	0.7969	0.8433	0.843	194
CR	0.9666	0.9658	0.8719	0.9165	0.905	201
SN	0.9801	0.9602	0.8673	0.9114	0.911	185
PD	0.9475	0.9588	0.9789	0.9687	0.901	217
CT	0.9857	0.9643	0.8529	0.9052	0.856	213
**Mean**	**0.9508**	**0.9501**	**0.8851**	**0.9159**	**0.8926**	**202.29**

Recg. Acc., Recognition Accuracy; ACT, Average Computational Time; SK, sky; BD, Building; RD, Road; SF, Side Fence; VG, Vegetation; PL, Pole; MK, Marking; CR, Car; SN, Sign; PD, Pedestrian; CT, Cyclist; Sec, Seconds.

#### 4.2.2 Comparison with existing methods

This section provides a comprehensive overview of the strengths and weaknesses when compared with other SOTA methods. During our experiments, it is witnessed an increase of a minimum of 6.4% in the accuracy of SUN-RGB-D while a 3.6% increase in the accuracy of NYU-Dv2 datasets is observed. Table 5 demonstrates the recognition accuracies over benchmark datasets for the proposed method along with other state-of-the-art techniques (Song et al., 2017) consider the spatial and semantic relationships between objects to enhance the discriminative power of scene recognition models. In Song et al. (2018) the authors aim to improve scene recognition performance by exploring various techniques for learning discriminative representations from RGB-D data. Additionally, they are offering insights into the importance of combining color and depth cues for effective scene recognition models (Du et al., 2019) the paper introduces the TRecgNet framework, which integrates cross-modal translation and modality-specific recognition tasks for scene recognition. By sharing a common encoder network and leveraging unlabeled data for translation training, TRecgNet improves the discriminative power of modality-specific recognition models. In Ayub and Wagner (2019) authors contribute a novel cognitively inspired clustering approach for RGB-D indoor scene classification. The method demonstrates state-of-the-art performance on benchmark datasets and offers insights into the space of centroids, leading to the proposal of a method for merging similar categories (Chen et al., 2018) present a reality-oriented adaptation approach for urban scene semantic segmentation using synthetic data. The approach addresses the challenges of overfitting and domain adaptation by learning real image style through distillation and aligning the distribution of synthetic and real domains.

**Table 5:** Comparison of recognition accuracies over SUN RGB-D and NYU=Dv2 datasets.

Method	SUN RGB-D	NYU-Dv2
Song et al. (2017)	–	66.9
Song et al. (2018)	53.8	67.5
Xiong et al. (2020)	56.2	68.1
Du et al. (2019)	56.7	69.2
Ayub and Wagner (2019)	59.5	70.9
Chen et al. (2018)	–	–
Gao et al. (2020)	–	–
**Proposed**	**94.27**	**72.80**

The SUN RGB-D and NYU-Dv2 datasets present unique challenges compared to the SYNTHIA dataset, primarily due to their differing environments and object characteristics.

These datasets consist of diverse indoor environments, including living rooms, kitchens, and offices, which introduce variability in lighting conditions and object occlusions. A wide range of object types with varying sizes and shapes need to be detected and recognized. Moreover, Indoor scenes often have high levels of clutter, leading to occlusion, which makes accurate object detection more challenging.

The dataset includes various outdoor conditions, such as different times of the day and weather conditions, affecting visibility and object appearance. Objects in outdoor environments can appear at a wide range of distances and scales, making it necessary for the detection model to generalize well across these variations. Outdoor scenes often have more complex and dynamic backgrounds, which can interfere with object detection accuracy.

The proposed method demonstrates superior performance across all datasets by effectively addressing these challenges through robust feature extraction and model adaptation techniques. The high accuracy achieved in both indoor and outdoor tasks highlights the versatility and efficiency of our approach.

#### 4.2.3 Statistical analysis

To ensure the superiority of the proposed approach (DFN) compared to the existing methods, various statistical tests are considered including paired t-test, ANOVA, etc. Here, we will use a paired t-test to compare the performance metrics of our proposed model with those of other state-of-the-art (SOTA) methods. We will perform paired t-tests comparing the mean accuracy of the DFN model against other methods.

Null Hypothesis (H0): There is no significant difference in performance between the DFN model and the compared methods.

Alternative Hypothesis (H1): The DFN model performs significantly better than the compared methods.

A significance level (α) is determined as 0.05 to accept or reject the hypothesis. If the *p*-value is less than (α), then the hypothesis will be rejected otherwise accepted. The detailed analysis is given in Table 6.

**Table 6:** Comparison of recognition accuracies over SYNTHIA dataset.

Method	SYNTHIA
Chen et al. (2018)	40.8
Gao et al. (2020)	80.0
**Proposed**	**95.08**

It is evident from Table 7, that all the *p*-values are less than 0.05. Hence the proposed DFN model performs significantly better than other state-of-the-art methods. This is demonstrated by the high t-statistics and low *p*-values from the paired t-tests, allowing us to reject the null hypothesis and conclude the superiority of the DFN model.

**Table 7:** Paired t-test results (recognition accuracy).

Dataset	*t*-value	*p*-value	Conclusion
SUN RGB-D	*t* = 7.15	*p* < 0.001	Reject H0 (significant)
NYU-Dv2	*t* = 4.32	*p* < 0.01	Reject H0 (significant)
SYNTHIA	*t* = 8.56	*p* < 0.001	Reject H0 (significant)

## 5 Discussion

The experimental results and subsequent analysis provide valuable insights into the effectiveness and robustness of the proposed DFN for contextual scene learning through multi-object detection and semantic analysis. The proposed DFN model demonstrates exceptional performance across all three benchmark datasets—SUN RGB-D, NYU-Dv2, and SYNTHIA. The mean accuracy achieved on these datasets is 94.27, 72.8, and 95.08%, respectively. These results underscore the robustness of the DFN model in handling diverse and complex scenes, both indoor and outdoor. The confusion matrices for the SUN RGB-D and NYU-Dv2 datasets reveal minimal misclassifications, indicating the model’s capability to accurately distinguish between various objects such as chairs, tables, sofas, walls, beds, bookshelves, and televisions. This precision in object recognition is crucial for applications requiring detailed scene understanding and reliable object detection.

These findings illustrate the superior performance of the DFN model across different datasets and scenarios, highlighting its ability to generalize well and outperform existing approaches. Moreover, to ensure the reliability of these results, paired t-tests were conducted to compare the performance metrics (mean recognition accuracy) of the DFN model against other SOTA methods. The *p*-values for all datasets are significantly less than 0.05, leading to the rejection of the null hypothesis. This statistical evidence confirms that the DFN model performs significantly better than other state-of-the-art methods. The high *t*-values and low *p*-values from the paired t-tests provide strong support for the superiority of the DFN model. The integration of RGB and depth information in the DFN model plays a critical role in enhancing scene understanding and object detection accuracy. By using a multi-modal fusion technique, the model may better utilize complementary information from many data sources, leading to an overall improvement in performance.

The proposed DFN model represents a significant advancement in contextual scene learning through multi-object detection and semantic analysis. The experimental results and statistical analysis validate its superior performance compared to existing methods. The high accuracy, minimal misclassifications, and adaptability across diverse scenarios underscore the robustness and reliability of the DFN model. These findings have broad implications for practical applications in computer vision, robotics, augmented reality, and autonomous systems, where precise scene understanding is crucial. Future research could further enhance the model by incorporating temporal and attention mechanisms, paving the way for even more sophisticated scene analysis and object detection solutions.

### 5.1 Model evaluation for object categorization

During training for object categorization, we employ the Adam optimizer with a learning rate of 0.001 and a batch size of 32. We use categorical cross-entropy as the loss function to measure the discrepancy between predicted probabilities and ground truth labels. The model is trained for 100 epochs, with early stopping based on the validation loss to prevent overfitting. We apply L2 regularization with a weight decay of 0.0001 to prevent excessive parameter growth. After training, we evaluate the MLP model on the testing split of the SUN RGB-D, NYU-Dv2, and SYNTHIA datasets. We compute various performance metrics, including accuracy, sensitivity, TPR, and F1 score, to assess the categorization performance. Additionally, we generate a confusion matrix to analyze the model’s ability to correctly classify objects into their respective categories. In the inference stage, we deploy the trained MLP model to categorize objects in new, unseen RGB-D images. The input image is preprocessed as described earlier, and the extracted features are passed through the MLP network. The output of the model corresponds to the predicted object category, providing valuable information for scene understanding and context-aware applications.

Hence, our proposed methodology for object categorization via MLP on the RGB-D datasets includes data preprocessing, feature extraction using a pre-trained VGG-16 model, MLP architecture design, training with specific parameter settings, evaluation on the testing dataset, and inference on unseen images is effective. The parameter settings, such as learning rate, batch size, and architecture configuration are incorporated as discussed earlier.

### 5.2 Model evaluation for contextual scene learning

When using FCNs for contextual scene learning based on object-to-object relationships over the RGB-D datasets, the following are the details of layers and parameter settings commonly used:

The encoder layers consist of convolutional layers followed by activation functions. The number of encoder layers can vary, but the configuration used here includes 10 convolutional layers with increasing numbers of filters (e.g., 64, 128, and 256). The filter size is set to 3×3, and the stride is set to 1. Padding will be used to maintain the spatial dimensions. For the down-sampling, 5 max-pooling layers are employed after certain encoder layers to reduce the spatial dimensions of the feature maps. The pool size is 2×2, and the stride is also set to 2 to achieve down-sampling by a factor of 2. The decoder layers aim to up-sample the feature maps to match the original input resolution. Transpose convolutional layers are commonly used in the decoder. The number of decoder layers matches the number of encoder layers, and the filter size, stride, and padding settings mirror the configuration of the corresponding encoder layers. Moreover, skip connections are used for integrating low-level and high-level features. These connections establish direct connections between the encoder and decoder layers to combine local and global contexts effectively. The various parameters used during the training are shown in Table 8.

**Table 8:** Parameter setting for contextual scene learning via FCN.

S. No.	Parameter	Value
1	Learning rate	0.01
2	Batch size	08
3	Epochs	50
4	Optimization algorithm	SGD
5	Loss function	Cross entropy loss + Dice loss

## 6 Conclusion

The paper presents a significant advancement in understanding contextual scenes through the application of Deep Fused Networks (DFN) for multi-object detection and semantic analysis. Our proposed approach strategically combines deep learning and fusion techniques to improve both accuracy and contextual understanding in complex scenes. The experimental results as well as the statistical analysis confirm the effectiveness of the method, demonstrating notable enhancements in object detection and semantic analysis compared to existing methods. The success of DFN suggests its potential in practical applications in computer vision, robotics, augmented reality, and autonomous systems. The improved accuracy in multi-object detection and semantic analysis has broader implications for tasks like autonomous driving, surveillance, and augmented reality applications, where precise scene understanding is crucial. Additionally, our findings underscore the significance of using deep learning and fusion techniques to address challenges posed by diverse scenes, occlusions, and object inconsistencies.

Looking forward, future research could explore incorporating temporal information to capture dynamic scene changes, introducing a temporal dimension to our contextual scene learning framework. Moreover, integrating attention mechanisms to selectively focus on relevant regions and objects within a scene represents a promising direction for enhancing the efficiency and adaptability of the proposed approach. These potential extensions aim to further advance the understanding of contextual scenes, providing valuable insights for researchers and practitioners.

## Data Availability

Publicly available datasets were analyzed in this study. This data can be found here: https://www.kaggle.com/datasets/thanhbnhphan/sun-rgbd-2d (SUN-RGBD 2D repository); https://www.kaggle.com/datasets/soumikrakshit/nyu-depth-v2 (Kaggle repository).
